# Supplementary material for: Unravelling reference bias in ancient DNA datasets
Source: Bioinformatics. 2024 Jul 3;40(7):btae436. doi: 10.1093/bioinformatics/btae436 (PMC11254355; doi:10.1093/bioinformatics/btae436)
Supplement: btae436_Supplementary_Data [file btae436_supplementary_data.zip › S1-S4_data_plots.zip!/S1-S4_data/rhino_sim_plots.zip!/rhino_sim_plots/bwamem_rhino_div1_1end_by_MQ.pdf]

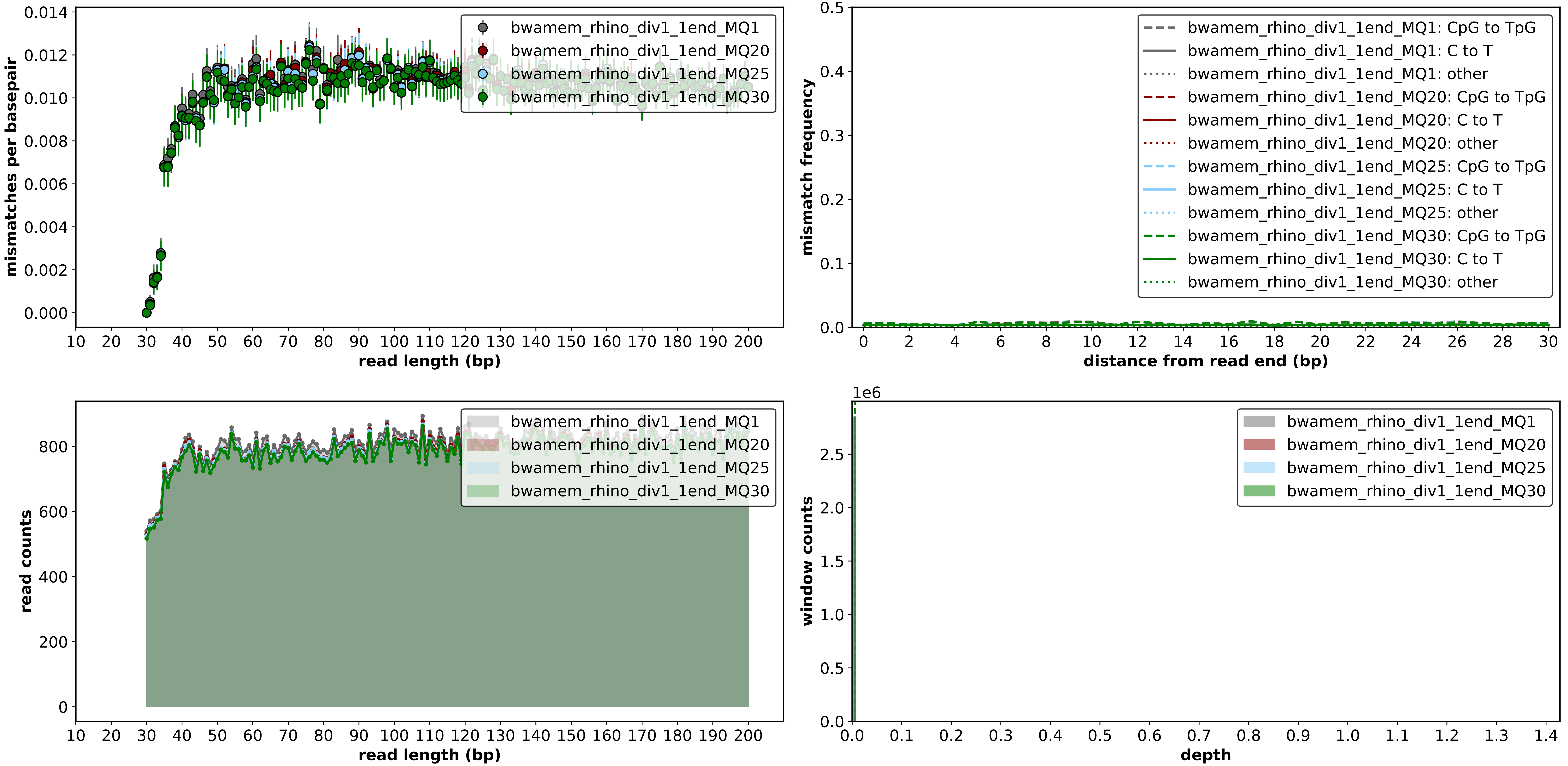
0.014
0.012
0.010
0.008
0.006
0.004
0.002
0.000
mismatches per basepair
10 20 30 40 50 60 70 80 90 100 110 120 130 140 150 160 170 180 190 200
read length (bp)
bwamem_rhino_div1_1end_MQ1
bwamem_rhino_div1_1end_MQ20
bwamem_rhino_div1_1end_MQ25
bwamem_rhino_div1_1end_MQ30
0.5
0.4
0.3
0.2
0.1
0.0
mismatch frequency
0 2 4 6 8 10 12 14 16 18 20 22 24 26 28 30
distance from read end (bp)
bwamem_rhino_div1_1end_MQ1: CpG to TpG
bwamem_rhino_div1_1end_MQ1: C to T
bwamem_rhino_div1_1end_MQ1: other
bwamem_rhino_div1_1end_MQ20: CpG to TpG
bwamem_rhino_div1_1end_MQ20: C to T
bwamem_rhino_div1_1end_MQ20: other
bwamem_rhino_div1_1end_MQ25: CpG to TpG
bwamem_rhino_div1_1end_MQ25: C to T
bwamem_rhino_div1_1end_MQ25: other
bwamem_rhino_div1_1end_MQ30: CpG to TpG
bwamem_rhino_div1_1end_MQ30: C to T
bwamem_rhino_div1_1end_MQ30: other
800
600
400
200
0
read counts
10 20 30 40 50 60 70 80 90 100 110 120 130 140 150 160 170 180 190 200
read length (bp)
bwamem_rhino_div1_1end_MQ1
bwamem_rhino_div1_1end_MQ20
bwamem_rhino_div1_1end_MQ25
bwamem_rhino_div1_1end_MQ30
1e6
2.5
2.0
1.5
1.0
0.5
0.0
window counts
0.0 0.1 0.2 0.3 0.4 0.5 0.6 0.7 0.8 0.9 1.0 1.1 1.2 1.3 1.4
depth
bwamem_rhino_div1_1end_MQ1
bwamem_rhino_div1_1end_MQ20
bwamem_rhino_div1_1end_MQ25
bwamem_rhino_div1_1end_MQ30
